# Gaze facilitates responsivity during hand coordinated joint attention

**DOI:** 10.1038/s41598-021-00476-3

**Published:** 2021-10-26

**Authors:** Nathan Caruana, Christine Inkley, Patrick Nalepka, David M. Kaplan, Michael J. Richardson

**Affiliations:** 1 Department of Cognitive Science, Macquarie University, 16 University Ave, Sydney, NSW 2109 Australia; 2 Perception in Action Research Centre, Macquarie University, Sydney, Australia; 3 Department of Psychology, Macquarie University, Sydney, Australia; 4 Centre for Elite Performance, Expertise and Training, Macquarie University, Sydney, Australia

**Keywords:** Sensorimotor processing, Sensory processing, Social behaviour, Social neuroscience

## Abstract

The coordination of attention between individuals is a fundamental part of everyday human social interaction. Previous work has focused on the role of gaze information for guiding responses during joint attention episodes. However, in many contexts, hand gestures such as pointing provide another valuable source of information about the locus of attention. The current study developed a novel virtual reality paradigm to investigate the extent to which initiator gaze information is used by responders to guide joint attention responses in the presence of more visually salient and spatially precise pointing gestures. Dyads were instructed to use pointing gestures to complete a cooperative joint attention task in a virtual environment. Eye and hand tracking enabled real-time interaction and provided objective measures of gaze and pointing behaviours. Initiators displayed gaze behaviours that were spatially congruent with the subsequent pointing gestures. Responders overtly attended to the initiator’s gaze during the joint attention episode. However, both these initiator and responder behaviours were highly variable across individuals. Critically, when responders did overtly attend to their partner’s face, their saccadic reaction times were faster when the initiator’s gaze was also congruent with the pointing gesture, and thus predictive of the joint attention location. These results indicate that humans attend to and process gaze information to facilitate joint attention responsivity, even in contexts where gaze information is implicit to the task and joint attention is explicitly cued by more spatially precise and visually salient pointing gestures.

Joint attention – the ability to share a common focus of attention with others – is fundamental to almost all cooperative human interactions^1^ and is foundational to the typical development of social cognition and language^2–6^. In a typical joint attention episode, one person *initiates* joint attention by looking towards, pointing at, or naming an object or event^7^. Their partner must then recognize this behaviour as communicative and respond to the joint attention bid by attending to the same location^8,9^.

To date, the empirical literature on joint attention has almost exclusively examined gaze-based social interactions, emphasizing the significance of social gaze as a rich source of social information^10–12^. This focus is motivated by the assumption that gaze has a unique dual function in which it can be used to both *signal* and *perceive* social information during interactions^13^. Furthermore, because shifts in gaze are both rapid and ubiquitous during face-to-face interactions, they have the capacity to provide continuous information about a social partner’s locus of attention and mental state^14^. However, these same characteristics also make social gaze a ‘noisy’ signal of intentional joint attention bids, since they are not always intentional, informative, or communicative^15^. For this reason, additional information, such as ostensive direct gaze (i.e., eye contact), are often needed during gaze-based interactions to convey the communicative intent of subsequent gaze shifts^16–19^. By contrast, situationally relevant actions, such as manual pointing gestures, provide less ambiguous joint attention signals because they are more spatially precise than gaze shifts and inherently convey communicative intent in most contexts^20–23^. This is because, pointing gestures, unlike gaze shifts, can extend into the exact location of the target – in many cases making direct physical contact – thus eliminating ambiguity concerning which specific object joint attention is being initiated towards.

In their recent theoretical and empirical work, Yu and Smith^23,24^ highlight how joint attention can be achieved using multiple non-verbal behaviours including gaze and pointing gestures. They found that while mothers achieved joint attention by shifting their gaze between the infant’s face, hands and target object, infants primarily tracked the goal-directed hand movements made by the mother, with limited attention to the mother’s gaze. These findings indicate that, at least during early development, processing gaze information is not essential for responding to joint attention bids. However, given that mothers did triadically attend to the face and hand-object dynamics of the infant, it remains unclear whether joint attention interactions require at least one person to be integrating gaze information to guide and evaluate whether joint attention has been achieved. Further work is needed to understand how *adult dyads* integrate and prioritize multimodal non-verbal information to achieve joint attention, since social information processing strategies are likely shaped by the developmental stage of both interacting individuals^5,8^.

Understanding which non-verbal behaviours humans rely on to achieve joint attention is important given that this affects how we conceptualize the cognitive mechanisms involved^9^. For instance, achieving joint attention by exclusively attending to hand-object dynamics might only rely on sensorimotor processes. This is likely to be less computationally demanding than exclusively relying on gaze signals which might require more abstract, social cognitive, inferences about their communicative value and specific spatial reference^24^. As such, to better characterize the mechanisms of joint attention, we must also consider how multiple communicative gestures are used together to achieve social coordination. This requires three key empirical blind spots to be addressed, including: (i) whether adults actually display useful gaze information when explicitly initiating joint attention via other gestures (e.g., hand pointing); (ii) the extent to which adults attend to the face of others before responding to joint attention bids; and (iii) whether relevant spatial information conveyed by the gaze of an initiator influences the efficiency with which a responder can achieve joint attention with their partner. These empirical blind spots exist due to a lack of experimental paradigms and technologies that can support realistic dyadic social interactions, whilst ensuring experimental control and objective measures of attention and socially responsive behaviours^10,25^. Several studies in the past decade have pioneered interactive paradigms for joint attention research, however the vast majority of these have exclusively examined gaze-based interactions^12,15,26–28^.

In the current study, we developed a novel interactive joint attention paradigm – employing immersive virtual reality, eye-, head- and hand-tracking technologies – to examine interactions between adult dyads where joint attention bids were initiated using pointing gestures. In each session, two participants sat opposite each other at a physical table in the laboratory. They were then immersed into a virtual laboratory that replicated the physical space using head-mounted displays with eye-tracking capability (Fig. 1). Each participant was represented by an avatar that they could control in real time by moving their eyes, head, and hands. Together, the dyad completed a cooperative search task which involved identifying a target amongst three objects placed on the virtual table. The target was visible to only one participant on each trial, with equal probability. The participant who located the target was the ‘initiator’ for that trial and was required to point to the correct target to initiate joint attention. The other person (i.e., the ‘responder’) was required to respond by also pointing to the correct target location. Critically, participants were explicitly instructed to use their hands to both initiate and respond to joint attention bids, but the use of one’s gaze was unconstrained in the task. This created a context in which gaze information could be used but was not necessary for task success. The integrated use of eye-tracking and immersive virtual reality provides two key advantages for investigating social information processing during joint attention. First, it allows for eye movement data to be examined without intrusive eye-tracking equipment obscuring the (virtual) face of each participant during the interaction. Second, it allows for eye movement data to be automatically and objectively segmented and analysed across dynamic areas of interest (e.g., the face of each avatar as participants move during and across trials) whilst being temporally-aligned to body movement data.

**Figure 1:**
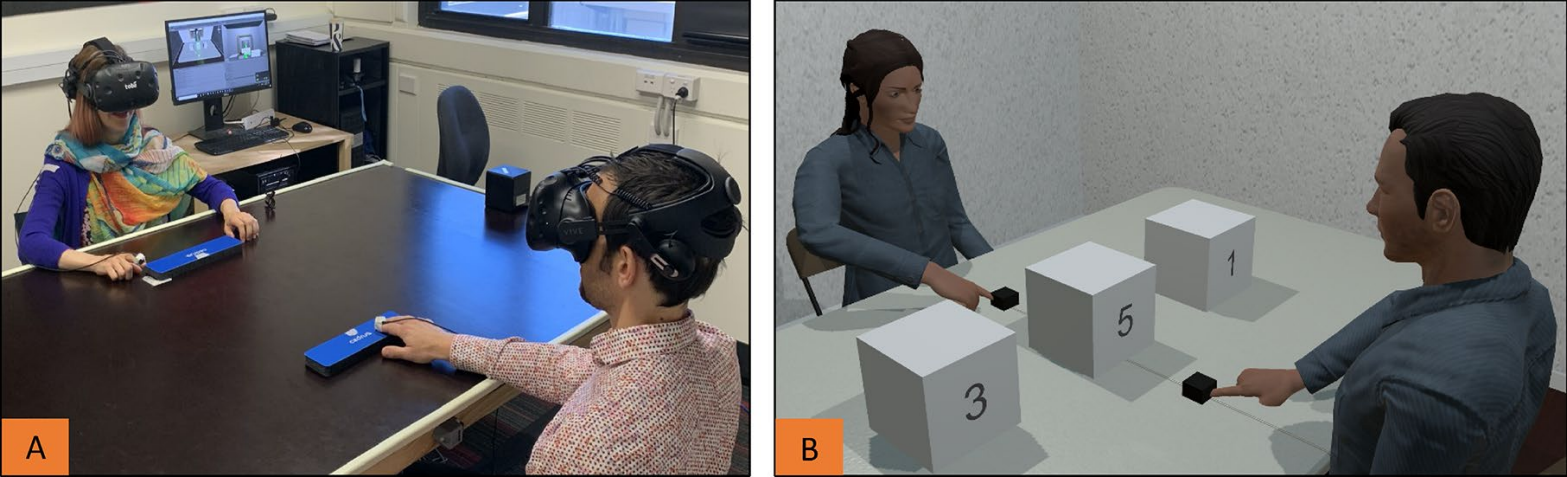
Dyads interacting in the (**A**) physical and (**B**) virtual laboratory. Note. Models photographed in 1A were not research participants from this study and provided written and informed consent for their photos to be used here.

Using this approach, we first wanted to investigate whether there was predictive information in the spatial pattern of an initiator’s gaze shifts before initiating joint attention via pointing. Evidence from reaching paradigms indicates that saccades to a visual target typically precede arm movements towards the same target^29,30^. This eye-hand coordination strategy affords the capture of detailed visual information from the fovea which can be used to improve the accuracy of subsequent hand movements^31^. We therefore expected initiators to gaze at the target location before pointing to it, thereby providing predictive spatial information about the upcoming joint attention bid.

Second, we wanted to investigate whether responders were influenced by this predictive gaze information, despite the availability of explicit pointing gestures. Previous gaze-only joint attention paradigms have found that people use spatial information conveyed by non-communicative eye-movements to facilitate responsivity to an upcoming gaze-cued joint attention bid^15,17^. Along the same lines, we predicted that responders would make faster saccades towards the target when initiator gaze and point behaviours were spatially congruent than spatially incongruent with each other. Next, we wanted to explore whether any observed effects of initiator gaze-point congruency on responsivity required responders to overtly attend to their partner’s face when joint attention was initiated.

Finally, we wanted to determine the extent to which participants spontaneously established eye contact before joint attention in our task, which did not force participants to communicate exclusively via social gaze. Previous studies of joint attention in gaze-based tasks have highlighted the importance of eye contact for facilitating joint attention responsivity^16,32^, presumably because eye contact provides an ostensive signal of communicative intent^15,17,18^. Given that pointing gestures inherently signal communicative intent, we anticipated that eye contact would rarely be established before achieving joint attention.

## Results

### Initiator gaze behaviour

**Figure 2:**
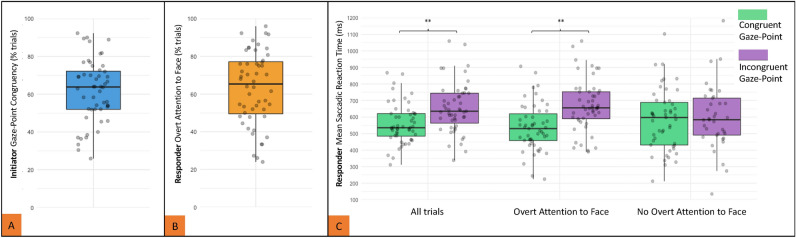
**(A)** displays the frequency of initiator gaze-point congruency across individuals (% trials); **(B)** displays the frequency of responder overt attention to the initiator’s face (% trials); **(C)** the effect of initiator Gaze-Point Congruency on SRT in milliseconds across all trials, on overt attention trials, and no overt attention trials. Data points represent individual means. ** p < 0.01.

First, we investigated whether participants displayed spatially congruent gaze behaviour prior to initiating joint attention by pointing. Specifically, we measured how often initiators directed their gaze towards the target before the onset of their pointing gesture. We found, on average, that initiators looked at the target immediately before pointing on only 51% of trials (*SD* = 19%; Fig. 2A). Overall, initiators were more likely to display congruent gaze when initiating joint attention towards a target located on the middle cube (79.8% congruent) than on one of the peripheral cubes (45.3% congruent). Interestingly, there was a high degree of variability between individuals, wherein the percent of trials with predictable gaze across participants ranged from 20–90% of initiator trials. These results indicate that the implicit gaze information displayed by initiators has the potential to guide joint attention responses. However, the reliability of this signal varies widely across individuals.

### Effect of initiator gaze behaviour on responsivity

Second, we tested whether responders extracted and used the implicit gaze information displayed by initiators to facilitate their joint attention responses. We operationalized this facilitation as faster saccadic reaction times (SRTs) by the responder when the initiator’s gaze and pointing behaviours were spatially congruent. As depicted in Fig. 2C, we found that responder saccadic reaction times were significantly faster on trials where the initiator’s gaze-point behaviours were spatially congruent (*M*_*SRT*_ = 618.45 ms; *SD*_*SRT*_ = 137.09 ms) than when they were incongruent (*M*_*SRT*_ = 674.97; *SD*_*SRT*_ = 176.63; *b* = 0.763, *SE* = 0.320, *t* = 2.385, *p* = 0.017). Model fit analysis (see methods for details) revealed that the addition of the congruency factor significantly improved the model’s fit to the available data 5.61 times better than the null model (*X*^2^(1) = 5.61, *p* = 0.017). Thus, this analysis revealed that variation in SRT data is better explained by a model which specifies variation in the congruency of the initiator’s gaze. This indicates that responders were influenced by the initiator’s gaze behaviour despite the availability of more explicit and obvious hand pointing joint attention gestures.

We also conducted an exploratory analysis to investigate whether the effect of initiator gaze-point congruency on responsivity was reliant on whether the responder was overtly attending to the initiator’s face. To test this, we first measured how often responders looked at the initiator’s face at the time of their pointing gesture. We found that, on average, responders overtly attended to their partner’s face on 60% (*SD* = 29%) of trials during this time (Fig. 2B). We then separately estimated model parameters for the gaze-point congruency effect twice: (i) only including the trials where responders overtly attended to the initiator’s face; and (ii) only including trials where responders did *not* overtly attend to the initiator’s face. Regarding (i), we found that when responders were overtly attending to the initiator’s face there was a significant effect of initiator gaze-point congruency on responder SRTs (see Fig. 2C). Specifically, SRTs were significantly faster when the initiator’s gaze was congruent (*M*_*SRT*_ = 635.29 ms; *SD*_*SRT*_ = 159.62 ms) rather than incongruent with their subsequent pointing gesture (*M*_*SRT*_ = 705.66 ms; *SD*_*SRT*_ = 223.56 ms; *b* = 1.293, *SE* = 0.385, *t* = 3.358, *p* = 0.001). In contrast, for analysis (ii), we found no evidence for a gaze-point congruency effect when responders did not overtly attend to the initiator’s face (*b* = 0.048, *SE* = 0.011, *t* = 0.094, *p* = 0.925). Model fits were then performed on data exclusively from trials in which responders overtly attended to the initiator’s face during joint attention. Compared to the null model, adding the congruency factor significantly improved the model fit by 11.01 times (*X*^2^(1) = 11.01, *p* < 0.001). These findings therefore suggest that the effect of initiator gaze-point congruency depends on the responder overtly attending to their partner’s face so that gaze informative can be processed.

### Frequency and effect of eye contact

Finally, the current study investigated how often dyads established eye contact before achieving joint attention. We found that, on average, dyads established eye contact prior to the initiator pointing at the target on as little as 4.08% of trials (*SD* = 7.98%). These results indicate that participants did not reliably use eye contact to signal an upcoming communicative exchange. An analysis of the effect of eye contact on joint attention responsivity was planned but not conducted given the low frequency of this behaviour.

## Discussion

Human gaze is undoubtably an important non-verbal signal used during reciprocal and coordinated social interactions. This notion has been supported by a large body of social neuroscience research over the past 40 years examining how humans observe and respond to *static* gaze information in non-interactive settings^5,14,33–36^. However, surprisingly little research has carefully examined how humans integrate and respond to gaze and other non-verbal behaviours (e.g., hand gestures) during *dynamic* and *reciprocal* social interactions. This is largely due to the practical and technological challenges of simulating complex social interactions, with multimodal forms of communication, whilst also maintaining experimental control and objectivity in the measurement of attention and behaviour^10^. To this end, the current study set out to develop a novel dyadic joint attention paradigm to investigate whether information embedded in the gaze of a social partner influences joint attention responsivity to joint attention bids initiated with a hand pointing gesture. Our paradigm combined immersive virtual reality, eye tracking and hand tracking technologies to: (i) enable participants to dynamically interact with their partner using eye, head, and hand movements in real-time; and (ii) objectively measure attention and social responsivity during joint attention interactions. We deployed this paradigm to ask three specific questions concerning the role of gaze during ecologically valid joint attention scenarios. First, do initiators of joint attention convey useful gaze information when intentionally using pointing gestures to guide their partner to the target location? Second, do responders use gaze information conveyed by initiators to facilitate their responsivity to joint attention bids – and to what extent does this rely on overt attention to the initiator’s face? Third, and finally, is eye contact important when joint attention is intentionally signaled using hand-pointing gestures?

### Initiator gaze behaviour

We anticipated that the gaze shifts made by initiators in the lead up to joint attention bids would convey predictive information about the locus of an upcoming joint attention target. Specifically, we expected initiators to gaze at the target immediately before pointing to it (i.e., display congruent gaze-point behaviour). If this were the case, it would suggest that initiator gaze – at the very least – has the *potential* to influence the joint attention responsivity of interlocutors. Consistent with this hypothesis, we found that initiators did fixate on targets immediately before pointing at them, however this was not always the case. Indeed, this gaze behaviour was highly variable across individuals and only occurred on approximately half of the trials. These findings deviate from hand-reaching paradigms, which consistently show that reaching for a target is reliably preceded by saccades toward the target location^29,30^. One possible explanation for our findings is that our task context reduced the need to use gaze at the start of a trial to refresh target location information before executing a motor action. Specifically, our task comprised three potential target objects which maintained a fixed location in the virtual environment. Given that participants also sat in the same position on all trials, it is highly plausible that participants employed a relatively stereotyped action plans for their pointing behaviours that made direct visual detection of the target’s location on every trial unnecessary. It is also possible that individual differences amongst initiators in the explicit use of communicative gaze signals may have influenced the extent to which their gaze was informative. For instance, if initiators were explicitly using gaze as a communicative signal, they would be more likely to look at the target before, or at least while, pointing. Conversely, those who were exclusively using their *pointing* gesture to communicate with their partner may have been less likely to exhibit informative gaze patterns if there was also no need for them to look at the target to plan their hand movements. This last explanation, however, seems unlikely, given that only four participants from different dyads (7.69% of the entire sample) indicated during the post-experimental interview that they were explicitly using gaze as a cue when initiating joint attention. When directly asked whether they preferred attending to hand or gaze information, only one of these participants, and six others (13.5% of the entire sample) indicated a preference for gaze. Taken together, it seems that very few participants were explicitly employing a gaze-specific strategy for either initiating or responding to joint attention bids. However, given that initiators, overall, displayed spatially congruent and incongruent gaze-point behaviours with almost equal probability, we were able to reliably test whether this impacted the responsivity of their partners.

### Effect of initiator gaze behaviour on responsivity

Critically, the current study predicted that when initiators exhibited congruent gaze-point behaviour, responders would be faster to saccade towards the target, compared to when the initiator’s gaze was incongruent with their point. In line with this prediction, we found that responders were faster to saccade towards the target when the initiator’s gaze was congruent than incongruent with their point. This suggests that humans have the capacity to integrate information conveyed by others’ gaze to facilitate joint attention responsivity, even when more spatially precise information (i.e., a pointing gesture) is available.

Importantly, across trials, there was a high degree of variability in whether responders overtly attended to (i.e., fixated on) the initiator’s face during joint attention (approximately 60% of the time). To determine whether this variability influenced the effect of initiator gaze on responsivity, we separately modelled the effect of initiator gaze-point congruency for trials where responders did and did not overtly attend to the initiator’s face during the execution of the joint attention bid. Consistent with our hypothesis, we found that the gaze-point congruency effect on responsivity remained significant when modelling data from trials where responders overtly attended to the initiator’s face, but not when modelling data from trials where they did not. Given that a large degree of variability was observed across individuals regarding the proportion of trials in which initiators displayed congruent gaze-point behaviours and the proportion of trials in which responders attended to their partner’s face, it is possible that the social information processing strategies employed by responders is influenced by the reliability of non-verbal signals displayed by initiators. For instance, interacting with a partner who reliably displays congruent gaze-point behaviour may encourage the responder to adopt a ‘face-looking’ and gaze integration strategy since the integration of gaze in this context, although more computationally demanding, would offer a reliable advantage in facilitating responsivity.

Relevant to the interpretation of ‘face-looking’ strategies is the fact that participants in our joint attention task were required to establish eye contact at the beginning of each trial. This task feature is consistent with previous experimental studies of joint attention responsivity (see Caruana et al., 2017 for a methodological review) and is essential in allowing each participant to signal their readiness to engage in the next trial without interrupting the reciprocal nature of the interaction with a salient environmental cue^10^. Eye contact is also an intuitive and ecologically-valid signal of communicative intent when initiating both non-verbal^15,17,19^ and verbal^18^ interactions. Nevertheless, it is possible that this task feature may have strengthened the tendencies of some participants to attend to their partner’s face during joint attention trials. However, such a bias does not appear to have manifested reliably across our sample given the high degree of variability across individuals in face-looking during joint attention (see Fig. 2B) and the almost complete absence of eye contact during trials (see below). Whilst it is of empirical value to examine the task and contextual factors that may influence face-looking tendencies in future research, this does not take away from the key finding here that humans have the capacity to use gaze information to facilitate joint attention responses – if and when they do attend to their partner’s face – despite the availability of more salient and spatially precise signals.

### Mutual eye contact

Eye contact has previously been proposed to offer an important gesture of communicative intent during non-verbal interactions due to its ability to first capture the attention of others and facilitate self-referential processing and attention allocation^14–16,26,33,34^. As such, we predicted that dyads would establish eye contact before pointing to initiate joint attention on most trials. We also expected that eye contact would lead to faster responses to joint attention bids compared to those with no eye contact. However, we found that dyads very rarely established eye contact before achieving joint attention in the context of our task. Overall, eye contact was only established on 4.08% of trials (*SD* = 7.98%). These findings reveal that eye contact is unlikely used, or indeed needed, to support joint attention responsivity when pointing gestures are available. This may be because pointing gestures, unlike other functional reach behaviours (e.g., grasping), are inherently communicative in most social contexts^32^. As such, eye contact provides no additional benefit to help responders identify whether their partner is intentionally initiating joint attention. This is often the case in gaze-only interactions where the communicative value of gaze is less clear^15^.

### Implications and future directions

The current findings suggest that there is marked variability in the extent to which initiators convey useful gaze information when joint attention is indicated explicitly using pointing gestures. There is also significant variation regarding whether responders overtly attend to the initiator’s face to extract gaze information during joint attention. Despite this variability, our data reveals that humans are nevertheless sensitive to the presence of informative gaze signals conveyed by others, which has a significant effect on their responsivity to joint attention opportunities. This is only the case when individuals overtly attend to their partner’s face. In this way, our data demonstrates that humans can, but do not reliably, integrate gaze and hand information during joint attention. We further propose that the variability observed in the current study may be the product of responders adopting different strategies for coordination depending on individual differences in the initiator’s signaling of reliable gaze information.

Future work using artificial agents whose gaze behaviour is precisely manipulable could directly confirm whether responders adaptively change their gaze integration strategies over time depending on the reliability (or volatility) of the initiator’s gaze. The novel paradigm established in the current study provides the field with a tool that can be used to test this directly in future studies by having participants interact with a pre-programmed agent (rather than another human, as in the current study) whose behaviour can be controlled and manipulated e.g.,^15,17,26^. This would enable the experimental manipulation of the avatar’s congruent gaze behaviour, as well as the volatility of this across trials. Such experiments would determine whether responders allocate less attention to—and are influenced less by—their partner’s gaze when the congruency of their partner’s gaze-point behaviours are more volatile throughout the interaction.

This avenue of future enquiry—and the potential for our paradigm—is also of great value to autism research, given that it is well established that autistic individuals display differences, and sometimes difficulties, in responding to joint attention and evaluating social information^36–38^. Moreover, in separate research, autistic individuals have been shown to adopt different patterns of predicting sensory events in their environment depending on the volatility of those events, compared to neurotypical individuals^39,40^. One possibility for the marked difficulties in navigating social interactions in autism may be that autistic individuals are less sensitive to identifying patterns of reliability and volatility in another person’s behaviour. If so, autistic individuals may be less likely to optimally adapt their social information processing strategy. The implementation of this novel paradigm for the investigation of social predictive coding and joint attention responsivity would be invaluable in testing this claim, informing a new model of social responsivity and information processing in autism.

Finally, it is important to recognize that this study presents only a first step towards understanding how humans integrate multiple channels of non-verbal information during joint attention. In order to better understand the factors that influence how and when we use gaze information, future research will need to focus on: (1) manipulating the gaze, hand and postural dynamics that might play a role in influencing joint attention responsivity (e.g., the temporal relationship between a gaze and pointing gesture); (2) expanding the range of verbal and non-verbal behaviours simultaneously available during interactions; and (3) moving towards more ecologically-valid, intuitive and diverse contexts for collaboration and social interaction.

## Conclusion

In the current study, a novel virtual reality paradigm was developed to investigate how humans integrate gaze shifts and pointing gestures during dyadic joint attention interactions. Specifically, we investigated the extent to which initiator gaze information was used by responders to guide joint attention responsivity in the presence of more visually salient and spatially precise pointing gestures. Our findings reveal that initiator gaze does contain predictive spatial information that can be used to guide the attention of responders. We also found that responders do overtly attend to the face of initiators before joint attention is achieved. However, both these initiator and responder behaviours were highly variable across individuals. Critically, we found that when responders did overtly attend to their partner’s face, saccadic reaction times were faster when the initiator’s gaze was congruent with spatial information conveyed by their pointing gesture. Interestingly, these effects were observed even though: (i) the predictability of the initiator’s gaze was often volatile across trials; (ii) the joint attention bid was explicitly signaled by a pointing gesture; and (iii) the majority of participants subjectively reported that they were preferentially attending to, and using, pointing gestures when responding to joint attention bids. This novel study provides the fields of social cognition and autism research with a paradigm that can be used to explore the mechanisms that support social coordination and communication, whilst maintaining ecological validity, experimental control and obtaining objective measures of social behaviour.

## Materials and methods

### Participants

Twenty-six dyads (n = 52, *M*_age_ = 19.65, *SD* = 2.61; 36 females) were recruited from undergraduate psychology units at Macquarie University and received course credit for their participation. All participants had normal or corrected-to-normal vision (i.e., with clear contact lenses only), had no history of neurological injury or impairment, and were right-handed as confirmed using the Edinburgh Handedness Inventory ^41^. All participants provided written informed consent prior to completing the study, and procedures were approved by the Macquarie University Human Research Ethics Committee. All experiments were conducted in accordance with relevant guidelines and regulations.

### Materials

#### Stimulus

A novel joint attention paradigm was developed for the present study using Unity3D Game Engine (Version 2017.4.19f.; San Francisco, CA, USA; See link for code https://github.com/ShortFox/Gaze-Responsivity-Hand-Joint-Attention). The virtual environment was designed to replicate the physical laboratory both in dimension (i.e., 3 m by 4.9 m room) and content (i.e., a table with two chairs on either side). The virtual table and chairs were isomorphic in size and location to their physical counterparts (i.e., table measured 1.5 m in length, 1.15 m in width, and 0.89 m in height; each chair had a seat height of 50 cm). To avoid metallic interference with the electromagnetic Polhemus motion-tracking system we used a purpose-built wooden table and polypropylene resin chairs.

Two anthropomorphic avatars (one female, one male) were created using Adobe Fuse CC (Beta Version 2014.3.14; San Jose, CA, USA) to represent the participants in the virtual environment. The female and male avatars differed in feminine and masculine features, respectively (e.g., slender or broad), however they were matched in clothing, skin tone, and low-level visual properties of the eyes (i.e., shape, size, colour, and location on face) to minimise visual differences. Additionally, the avatars were designed to be ethnically ambiguous in appearance (i.e., a composite of features that did not clearly represent a specific racial group). An independent sample of 47 participants (M_age_ = 37.28, SD = 10.33, 31 females) were asked to identify the ethnicity of each avatar and rate how certain they were of their response on a 7-point Likert scale (1 = not at all; 7 = completely). All participants indicated a high degree of uncertainty. Only 2.1% and 6.4% of participants indicated that they felt completely confident about the ethnicity of the female and male avatar respectively. This was to mitigate any potential ‘other-race’ or out-group biases impacting on social interaction behaviour^42^.

#### Apparatus

The virtual environment was presented to participants via a virtual reality head-mounted display (HMD) with a 105° vertical and 94° horizontal field of view (HTC Vive, HTC Corporation, Taiwan; Valve Corporation, Bellevue, Washington, USA). The two HMDs used for the study (one for each participant in a pair) were retrofitted with an eye-tracker (Tobii Pro Inc., Sweden), such that it recorded the participant’s eye movements (i.e., saccades, fixations, and blinks) as well as head movements (i.e., angle and position). This was used to measure each participants’ gaze behaviour, but also to control the gaze dynamics of each participant’s virtual avatar, which could be seen by their partner during the task. Right index finger movements (i.e., position, orientation, and trajectory) were recorded via a Polhemus G4 tracking system (Polhemus Inc, Vermont, USA) which provided six degrees of freedom in motion tracking with a sampling rate of 120 Hz. A plugin provided by Polhemus was used to interface the motion tracking system with Unity3D. The Polhemus motion sensor data was used to control the avatar’s right index finger ^43,44^. Given that only finger motion – and not whole arm motion – was measured, we used an inverse kinematics calculator (RootMotion Inc., Estonia) to realistically generate the remaining full-arm and upper torso movement^43,44^. The maximum display latency between the two participant’s real-world movements and their movements in the virtual environment was 33 ms.

The stimuli presented within the virtual environment were overlayed with analysis-related areas of interest (AOIs) to record when a participant viewed these regions during the course of the task (i.e., gaze, hand, body, cubes, and other). A look to their partner’s face was recorded when a participant’s view came within 7.5° of visual angle from their partner’s nasion (i.e., point between the eyes and above the nose). This criterion was chosen as human acute vision has a span of approximately 15° of visual angle ^45^. Therefore, fixations within a 7.5° radius of the nasion can still extract information from the avatar’s eye gaze (see Supplementary Material 2 for a visualized AOI). Following peer review, we repeated all analyses using a smaller AOI around the eye region only. This produced the same pattern of results reported in the manuscript. The detailed analysis is reported in a separate RMarkdown on this study’s OSF page (see Data and Statistical Analysis, below).

A look towards a partner’s pointing hand was recorded when the participant fixated within the ‘hand’ AOI (10 × 20 cm), which began at the avatar’s right wrist and extended to the index finger. A look towards their partner’s body was recorded when a participant’s gaze fell within the ‘body’ AOI (40 × 60 cm)*.* This area began at the avatar’s hips, spanned the width of the body, and ended at the base of the neck. An AOI was also defined for each of the three task-relevant cubes, which aligned with the parameters of each cube (10 × 10 × 10 cm).

### Procedure

#### Set up

Two participants were recruited for each session. Once both had arrived, they were invited into the laboratory and were seated in chairs directly across from one another at a table. Once all relevant paperwork was completed, task instructions were provided to both participants simultaneously. To ensure that participants engaged in the task as naturally as possible, specific instructions on how to interact with each other were not provided, and care was taken not to mention gaze as a focus of investigation in the current study. This was critical in mitigating any bias of attention to the face or eyes during the task. The experimenter then attached a motion sensor to each participant’s right index finger using masking tape and secured the virtual reality HMDs onto each participant’s head which was adjusted to ensure visual acuity and optimal convergence given the distance between the participant’s eyes.

Based on the demographic information provided at the beginning of the experiment, participants were assigned an avatar that matched their own self-declared gender. An eye-tracking calibration was then conducted to align the first-person view of the participant with their avatar’s in the virtual space. Participants were then able to view the virtual environment for the first time from the perspective of their avatar; however, they could not yet see their partner. Instead, a mirror was positioned on the wall in front of each avatar to allow participants to observe their virtual body and to promote embodiment of the virtual body as they viewed the congruent visual-motor feedback while they moved their hand, head, and eyes. This was also an opportunity to demonstrate the interface’s capacity to accurately record and display their body movements in real time. At this point, both participants saw a row of three white cubes placed on the table in front of them (10 × 10 × 10 cm), positioned 30 cm apart and an equal distance between both participants. Participants were informed that these cubes would be the focus of their search during both the non-social and social tasks.

#### Non-social task

Alone in the virtual environment, the two participants first completed 57 non-social search trials on their own. This task was added to mitigate novelty effects associated with being in virtual reality. The non-social trials also provided an opportunity to check that both the eye and hand tracking technology was adequately calibrated before the social task commenced. Finally, these trials enabled us to measure variability in saccadic reaction times in a similar, yet non-social task context both when a hand pointing response was and was not required. This was primarily motivated by our intention to apply this task in future studies comparing joint attention responsivity between autistic and non-autistic individuals. This will help elucidate whether any observed differences are unique to the social domain, or relate to domain general processes including oculomotor control or attention. To this end, participants completed two sets of non-social trials. First participants completed 27 saccadic-response trials, and then 27 consecutive point-response trials. See Supplementary Material 1 for a full description of the non-social task as well as a summary statistics for SRTs observed across these tasks.

#### Joint attention task

**Figure 3:**
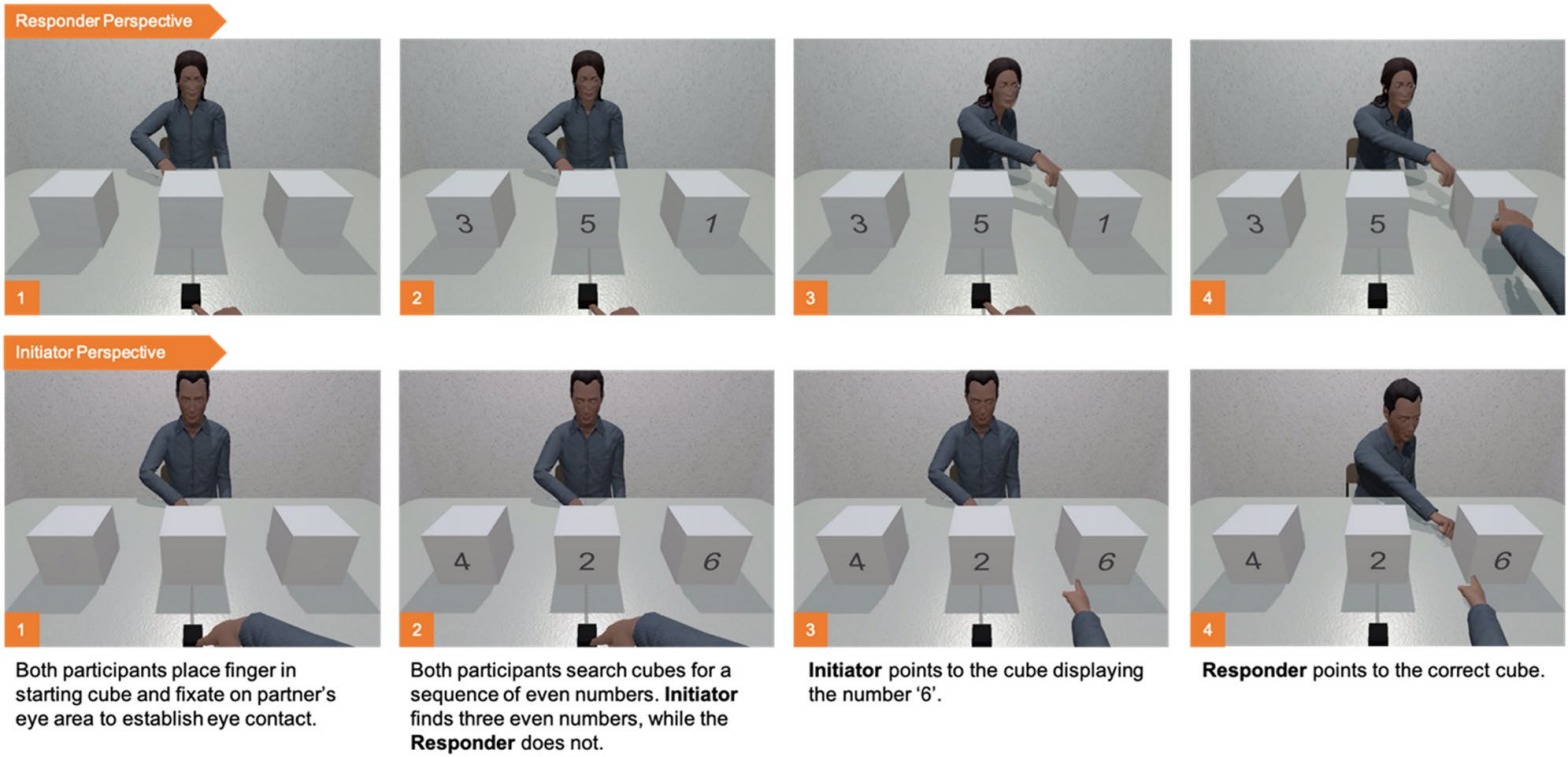
Example trial sequence representing the joint attention task from the perspective of the Initiator and Responder.

Following the non-social task, participants completed the joint attention task, which comprised the trials of interest in the current study. Unlike the non-social tasks which required participants to work alone in their virtual environment, the joint attention task allowed the participant dyad to share the same virtual space, where they could observe and interact with each other. The joint attention task required the dyad to engage in a simultaneous search, the outcome of which would implicitly inform each participant’s role as either the *initiator* or *responder* of joint attention for that trial. Participants would carry out their role, and thus achieve joint attention, by using a pointing gesture (see Fig. 3 for a representation of the trials sequence and the SI Video for an example of Congruent and Incongruent Gaze-point Initiator behaviour as seen from the perspective of both the Initiator and Responder).

The trial began when both participants had placed their right index finger into the starting cube (5 × 5 × 5 cm) and fixated on their partner’s face (see AOI definition above). Once mutual eye contact had been established, the participants would engage in a simultaneous search by inspecting the numbers presented to them on the three white cubes. On 50% of the trials, Participant A was presented with three even numbers (i.e., 2, 4, 6), while Participant B was presented a mix of odd and even numbers (e.g., 5, 2, 1). On these trials, Participant A would act as the initiator of joint attention and was required to direct Participant B, using a point gesture, to the cube with the highest number (i.e., this would always be a ‘6’). Participant B, acting as the responder, was instructed to follow Participant A to the target cube by also pointing to the correct cube. On the remaining 50% of the trials, Participant B would take the role of the initiator, assigned by the presentation of three even numbers, and consequently Participant A would act as the responder. In total participants completed 54 joint attention trials (27 as initiator, 27 as responder). Trials were presented in randomized orders, with target location, cube number combinations and number location counterbalanced across trials. Participants were instructed to complete the task as quickly and as accurately as possible.

#### Feedback

Following the successful fixation on the target (saccade-response trials) or point towards the target (point-response trials, joint attention task), the correct cube would turn green. Alternatively, if the participants pointed at a non-target cube, the cube would turn red to indicate an incorrect search. The same trial feedback was provided for both non-social and joint attention task trials.

### Behavioural measures

#### Initiator behavior

Initiating a joint attention bid was defined in the current paradigm as the communicative point towards the target made by the participant acting as the initiator. Onset latency (rather than the execution time) was measured because it afforded natural variance in the duration of point trajectories – due to varied cube distance, arm length, or ending location on cube. The movement onset was calculated as the point in time when the speed of the initiator’s hand towards the targeted object was at least 5% of the maximum speed value obtained from the trial. This 5% velocity threshold was chosen in order to exclude non-informative movements towards non-targeted objects, whilst including the earliest time that directional information could be conveyed by the movement ^46^.

We also recorded the order in which initiators searched all three locations before pointing to the target cube. In this way, we were able to assess whether the initiator’s gaze direction was congruent with their point location. Initiator gaze was defined as congruent when the location fixated immediately before the joint attention bid (i.e., point movement onset) was the target cube location. If the initiator fixated on a non-target cube immediately prior to pointing, this was considered a non-congruent gaze shift. However, if the initiator looked at the target cube and then to the eyes of their partner before initiating joint attention, this sequence was considered ‘uncharacterizable’ in terms of gaze-point congruency. However, this sequence of initiator eye movements did provide an opportunity for eye contact prior to the onset of the joint attention bid, the effect of which we examined separately.

For SRT measures, a saccadic response was defined as the first look towards the target cube after the initiator’s point movement onset (as defined above). Responder SRTs were calculated as the amount of time that elapsed between initiator point onset and the onset of the responder’s subsequent saccade towards the target cube. Consistent with our previous SRT analyses, responder saccades towards the target that occurred prior to 100 ms after the point onset were excluded as they were likely to be anticipatory responses or false starts^15^.

### Data and statistical analysis

#### Pre-processing data

During the task, the position and timing of each participant’s eye gaze, hands and head movements were continuously recorded at 60 Hz. Interest area output and trial data were exported from Unity3D using a custom Matlab script (MATLAB R2017b; See link for code https://github.com/ShortFox/Gaze-Responsivity-Hand-Joint-Attention), then screened and analysed in R 3.6.1 using a custom RMarkdown script (R Core Team, 2019–07-05; see RMarkdown document at https://osf.io/pfx4k/?view_only=7495d0a1f00d44f6820e8e30738fcaa5 for a detailed description of each processing step and all analysis output). Following data screening, all 52 participants were retained; however, 208 trials were excluded due to incorrect responses or poor eye-tracking calibration. On average participants responded incorrectly (i.e., pointed to a non-target cube) on 2.0% of trials (*SD* = 2.6%), resulting in 60 trials being removed overall. However, due to the rarity of their occurrence, no error analysis was explored on these trials. The validity of eye calibration fell below 90% on 54 trials, and thus they were removed from analyses. Moreover, consistent with our previous work, trials in which responder SRTs were longer than 3000 ms were excluded, as they were unlikely to reflect an immediate response to the joint attention bid^15^. A square root and a log_10_ transformation were then performed on saccadic reaction time data and point reaction time data, respectively, to meet the distributional assumption of linear mixed effects (LME) models, as skewed data can bias the estimation of model parameters^47^. The specific transformation used was objectively determined using the box-cox function in R^48^.

#### Linear mixed effects analysis

The effects of initiator gaze-point congruency on joint attention responsivity were statistically analysed by estimating LME models, using the maximum likelihood estimation method within the *lme4* R package^49^. A linear mixed effects model was employed instead of a traditional analysis of variance (i.e., ANOVA), in order to account for subject, pair and trial-level variance (i.e., random effects) when estimating the fixed effect parameter (i.e., gaze-point congruency). Studying dyadic interactions presents the challenge of appropriately accounting for the random variance associated with each participant, as well as the interacting partner they are paired with. An LME model more accurately captures the interdependent nature of the current interactive task, by providing a structure that nests the random effects of each subject within the random effects of the pair they belong to (coded as “pair/subject”). Furthermore, given that the variables of interest (e.g., congruent gaze, eye contact) are naturally occurring in our paradigm rather than enforced by the task, it was anticipated that we would not obtain a balanced number of observations (e.g., for congruent or incongruent gaze-point sequences) for each subject or pair. The LME approach accounts for this by treating each trial, rather than each subject, as a unique observation^50,51^. As such, because LME analyses do not rely on aggregated measures (i.e., averaged measures across trials per subject and condition) they are robust to missing data and unbalanced observations.

In line with recommendations for implementing mixed random-effects models, we adopted a “maximal” random factor structure, with random intercepts for subject/pair, and trial^52^. Specifically, we originally defined a saturated model including random intercepts for the trial by pair/subject factors. However, the “maximum likelihood” of this complex model could not be estimated given the available data and “failed to converge”^52^. Therefore, we simplified our random-effects parameters to define the most saturated yet parsimonious model. The simplified model included random intercepts for the trial by pair/subject factors. P-values were estimated using the *lmerTest* package^53^, and a significance criterion of α < 0.05 was employed.

To quantify the variance explained by our fixed effect factor, we compared two mixed random-effects models^54^. The first model comprised of the random effects only (i.e., subject, pair, trial), and the second mixed random-effects model included these random effects as well as our fixed factor (i.e., gaze predictability). A chi-square likelihood ratio was calculated for the comparison to determine the extent to which the fixed effect parameter improved model fit, and whether that improvement was significant. These analyses are presented in lieu of traditional effect size statistics (e.g., R-square), which are unable to account for the variance explained by each fixed effect, over-and-above variance already explained by the defined random effects.

### Data availability

All data used in this study have been de-identified and made available on the Open Science Framework (https://osf.io/pfx4k/?view_only=7495d0a1f00d44f6820e8e30738fcaa5 ).

### Code availability

A novel joint attention paradigm was developed for the present study using Unity3D Game Engine (Version 2017.4.19f.; San Francisco, CA, USA). Interest area output and trial data were exported from Unity3D using a custom Matlab script (MATLAB R2017b). Both sets of code are available on GitHub (https://github.com/ShortFox/Gaze-Responsivity-Hand-Joint-Attention). Exported data were then screened and analysed in R 3.6.1 using a custom RMarkdown script (R Core Team, 2019–07-05; see RMarkdown document at https://osf.io/pfx4k/?view_only=7495d0a1f00d44f6820e8e30738fcaa5 for a detailed description of each processing step and all analysis output).

## Supplementary Information

Supplementary Information 1.

Supplementary Information 2.

Supplementary Information 3.

Supplementary Video 1.
