# Supplementary material for: Gaze facilitates responsivity during hand coordinated joint attention
Source: Sci Rep. 2021 Oct 26;11:21037. doi: 10.1038/s41598-021-00476-3 (PMC8548595; doi:10.1038/s41598-021-00476-3)
Supplement: Supplementary file 1 — Supplementary Information 1. [file 41598_2021_476_MOESM1_ESM.zip › OSF_DataAnalysis_Revision/AvatarValidation/Avatar Validation Questionnaire.pdf]

Avatars

This is a very short survey to assess how people perceive our new avatar stimuli.

* Required

1

What is your age (years)? *

2

What is your gender? *

- Male
- Female
- Prefer not to say
- Other

3

What ethnicity do you identify with? *

- Caucasian
- Hispanic
- South Asian (e.g., Thai, Vietnamese, Malaysian)
- East Asian (e.g., Indian, Sri Lankan, Nepalese)
- African
- Middle Eastern
- Pacific Islander
- Other

4

What ethnicity do you think this female avatar is? *

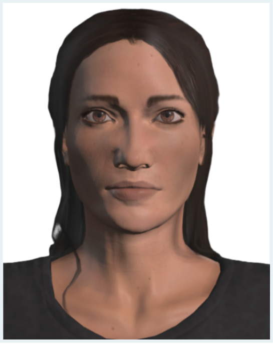

- ◯ Caucasian
- ◯ Hispanic
- ◯ South Asian (e.g., Thai, Vietnamese, Malaysian)
- ◯ East Asian (e.g., Indian, Sri Lankan, Nepalese)
- ◯ African
- ◯ Middle Eastern
- ◯ Pacific Islander
- ◯ Other

5

How certain are you about the female avatar's ethnicity? *

	Not at all			Somewhat			Completely
How certain?	◯	◯	◯	◯	◯	◯	◯

6

What ethnicity do you think this male avatar is? *

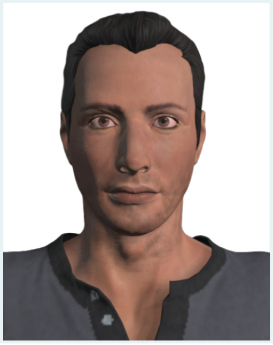

- ◯ Caucasian
- ◯ Hispanic
- ◯ South Asian (e.g., Thai, Vietnamese, Malaysian)
- ◯ East Asian (e.g., Indian, Sri Lankan, Nepalese)
- ◯ African
- ◯ Middle Eastern
- ◯ Pacific Islander
- ◯ Other

7

How certain are you about the male avatar's ethnicity? *

	Not at all			Somewhat			Completely
How certain?	◯	◯	◯	◯	◯	◯	◯

This content is neither created nor endorsed by Microsoft. The data you submit will be sent to the form owner.

Microsoft Forms
