# Supplementary material for: Gaze facilitates responsivity during hand coordinated joint attention
Source: Sci Rep. 2021 Oct 26;11:21037. doi: 10.1038/s41598-021-00476-3 (PMC8548595; doi:10.1038/s41598-021-00476-3)
Supplement: Supplementary file 2 — Supplementary Information 2. [file 41598_2021_476_MOESM2_ESM.docx]

Supplementary Information for:

**Gaze Facilitates Responsivity During Hand Coordinated Joint Attention**

Nathan Caruana* ^1^, Christine Inkley* ^1^, Patrick Nalepka ^2 3 4^, David M. Kaplan^1 2 4^, & Michael J. Richardson^2 3 4^

*****These authors contributed equally to this manuscript.

*^1^ Department of Cognitive Science, Macquarie University, Sydney, Australia*

*^2^ Perception in Action Research Centre, Macquarie University, Sydney, Australia*

*^3^* *Department of Psychology, Macquarie University, Sydney, Australia*

*^4^* *Centre for Elite Performance, Expertise and Training, Macquarie University, Sydney, Australia*

**Corresponding author:**

Nathan Caruana

Department of Cognitive Science, Macquarie University

16 University Ave, Macquarie University, Sydney, NSW 2109, Australia.

**Tel:** +61 2 9850 2989

**E-mail:** [nathan.caruana@mq.edu.au](mailto:nathan.caruana@mq.edu.au)

**Supplementary Information 1: Description of Non-Social Virtual Reality Tasks**

The two participants first completed 57 non-social search trials on their own. Each participant completed the task alone, in their own (separate) virtual environment. First participants completed 27 saccadic-response trials, and then 27 consecutive point-response trials. These trials only required participants to respond to spatial cues and did not require them to search for or identify numerical targets as in the joint attention task.

**Saccadic-response trials.** The saccadic-response trials required participants to respond to a peripheral cue by looking at the correct cube only. These trials began with the participant fixating on a black rectangle (15 x 10 cm) that was located on a virtual wall in front of them. Functioning as a fixed starting point for the participant’s gaze, the position of the rectangle was matched to the height of their partner’s eye region. Once participants had fixated on the rectangle for 3000 ms the rectangle turned grey, and after a further random delay of 1000-2000 ms, one of the three white cubes turned yellow to cue participants to this location. Participants were then required to direct their eye gaze towards the highlighted target cube. Saccadic reaction times were calculated as the time between the yellow cue appearing and the onset of the participant’s saccade towards the correct target. Saccadic reaction times on this task were generally faster and less variable than those observed in the joint attention task (see Figure 1A., *M* = 401.46, *SD* = 136.22).

**Point-response trials*.*** The point-response trials required participants to respond to an equivalent cue by pointing to the target. Each trial began with participants placing their right index finger into a starting cube (5 x 5 x 5 cm) positioned 30 cm in front of them on the table in the virtual space. Participants then fixated on the black rectangle that was located on a virtual wall in front of them. Once participants had fixated on the rectangle for 3000 ms, the rectangle turned grey and after a further random delay of 1000-2000 ms, one of the three white cubes turned yellow to cue participants to this location. Participants were then required to point towards the highlighted target cube. Saccadic reaction times on this task were calculated in the same way as the saccadic-response trials and were also found to be generally faster and less variable than those observed in the joint attention task (see Figure 1B., *M* = 502.03, *SD* = 177.11).

|  |
| --- |
| **Figure 1.** SRTs in milliseconds for **(A)** Saccadic Response Non-social task, **(B)** Point-response Non-social task, and **(C)** for both Congruent and Incongruent Responding trials on the Joint Attention task. Data points represent individual means. ** p < 0.01. |
