# Supplementary material for: Gaze facilitates responsivity during hand coordinated joint attention
Source: Sci Rep. 2021 Oct 26;11:21037. doi: 10.1038/s41598-021-00476-3 (PMC8548595; doi:10.1038/s41598-021-00476-3)
Supplement: Supplementary file 3 — Supplementary Information 3. [file 41598_2021_476_MOESM3_ESM.docx]

Supplementary Information for:

**Gaze Facilitates Responsivity During Hand Coordinated Joint Attention**

Nathan Caruana* ^1^, Christine Inkley* ^1^, Patrick Nalepka ^2 3 4^, David M. Kaplan^1 2 4^, & Michael J. Richardson^2 3 4^

*****These authors contributed equally to this manuscript.

*^1^ Department of Cognitive Science, Macquarie University, Sydney, Australia*

*^2^ Perception in Action Research Centre, Macquarie University, Sydney, Australia*

*^3^* *Department of Psychology, Macquarie University, Sydney, Australia*

*^4^* *Centre for Elite Performance, Expertise and Training, Macquarie University, Sydney, Australia*

**Corresponding author:**

Nathan Caruana

Department of Cognitive Science, Macquarie University

16 University Ave, Macquarie University, Sydney, NSW 2109, Australia.

**Tel:** +61 2 9850 2989

**E-mail:** [nathan.caruana@mq.edu.au](mailto:nathan.caruana@mq.edu.au)

**Supplementary Information 2: Face Area of Interest**

| 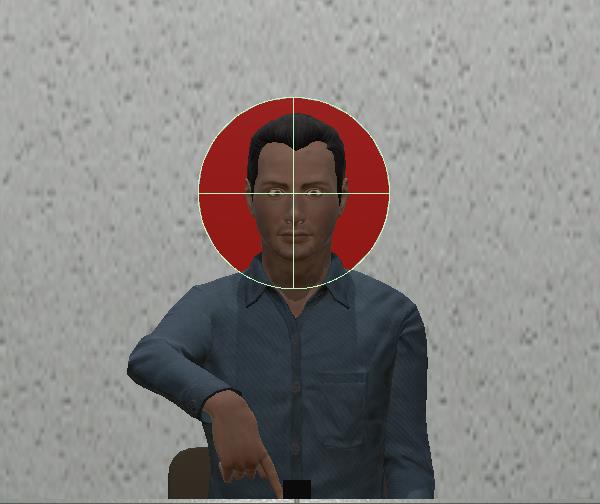 |
| --- |
| **Figure 1.** Red circle represents the ‘face’ AOI. A look to their partner’s face was recorded when a participant’s view came within 7.5° of visual angle from their partner’s nasion (i.e., point between the eyes and above the nose). |
